# Cholesterol as a modifying agent of the neurovascular unit structure and function under physiological and pathological conditions

**DOI:** 10.1007/s11011-017-0015-3

**Published:** 2017-04-21

**Authors:** Ewelina Czuba, Aleksandra Steliga, Grażyna Lietzau, Przemysław Kowiański

**Affiliations:** 1 Department of Anatomy and Neurobiology, Medical University of Gdańsk, 1 Dębinki Str, 80-211 Gdańsk, Poland; 2 Department of Health Sciences, Pomeranian University of Słupsk, 64 Bohaterów Westerplatte Str, 76-200 Słupsk, Poland

**Keywords:** Alzheimer’s disease, Blood brain barrier, Cholesterol homeostasis, Neurodegeneration, Neurovascular unit, Oxysterols

## Abstract

The brain, demanding constant level of cholesterol, precisely controls its synthesis and homeostasis. The brain cholesterol pool is almost completely separated from the rest of the body by the functional blood-brain barrier (BBB). Only a part of cholesterol pool can be exchanged with the blood circulation in the form of the oxysterol metabolites such, as 27-hydroxycholesterol (27-OHC) and 24S–hydroxycholesterol (24S–OHC). Not only neurons but also blood vessels and neuroglia, constituting neurovascular unit (NVU), are crucial for the brain cholesterol metabolism and undergo precise regulation by numerous modulators, metabolites and signal molecules. In physiological conditions maintaining the optimal cholesterol concentration is important for the energetic metabolism, composition of cell membranes and myelination. However, a growing body of evidence indicates the consequences of the cholesterol homeostasis dysregulation in several pathophysiological processes. There is a causal relationship between hypercholesterolemia and 1) development of type 2 diabetes due to long-term high-fat diet consumption, 2) significance of the oxidative stress consequences for cerebral amyloid angiopathy and neurodegenerative diseases, 3) insulin resistance on progression of the neurodegenerative brain diseases. In this review, we summarize the current state of knowledge concerning the cholesterol influence upon functioning of the NVU under physiological and pathological conditions.

## Introduction

Cholesterol is an imperative factor in the human central nervous system (CNS) and plays a crucial role in regulation of both physiological and pathological processes. Among the first, the most important functions of cholesterol are associated with the maintenance of the optimal level of energetic metabolism, the composition of cell membranes and myelination. Among the second, the most important issues comprise metabolic disorders leading to the development of Alzheimer’s (AD), demyelination and consequences of cerebral ischemia. The role of cholesterol metabolism in the CNS has to be considered taking into account its main components: neurons, cerebral blood vessels and neuroglia. The results of structural and functional studies indicate a precise interdependence of these three components, which is reflected in the concept of the neurovascular unit (NVU) (Kowiański et al. 2013). Although this concept undergoes constant evolution, which is reflected in the different number of its constituents accepted by numerous authors, it is commonly accepted that the most important components of this functional unit are: the neurons, the vascular endothelial cells, the smooth muscle cells of the vascular wall, the pericytes, the astrocytes and the perivascular macrophages (Abbott et al. 2006; Zlokovic 2011; Kowiański et al. 2013; Hawkes et al. 2015). Recent studies indicate the pathogenic role of vascular macrophages in NVU dysfunction and indicate their contribution to cognitive dysfunction in hypertension by their effect on the increase of BBB permeability in murine models (Faraco et al. 2016). Analysis of the cholesterol metabolic processes in the brain under physiological conditions, must therefore take into account the close cooperation between all of NVU’s components.

### Brain cholesterol metabolism in physiological conditions

Despite the fact that the human brain consists only 2% of the entire body mass, it contains a quarter of the non-esterified cholesterol pool, making it the most cholesterol-rich organ (~23 mg/g of tissue) in the body (Dietschy and Turley 2004). In the adult brain a significant part of cholesterol pool is concentrated in the myelin of white matter fibers, which contains up to 70% of the total pool (40 mg/g) (Russell et al. 2009), whereas the remaining part - in the plasma membrane of neurons and glial cells (Dietschy and Turley 2004; Orth and Bellosta 2012). This pattern of cholesterol distribution reflects its significance for the processes of electrical excitation of neurons, synaptic transmission and maintaining the nervous tissue morphology (Dietschy and Turley 2004). The rate of cholesterol synthesis is correlated with the stage of brain development and its level varies in different brain regions (Morell and Jurevics 1996; Saher et al. 2005). Consequently, the highest rate of cholesterol synthesis is observed during myelination of the white matter structures. In the adult brain, cholesterol synthesis decreases considerably, remaining at a low level (Morell and Jurevics 1996; Saher et al. 2005).

#### Autonomy of the brain cholesterol synthesis is determined by functional barriers

**Fig. 1:**
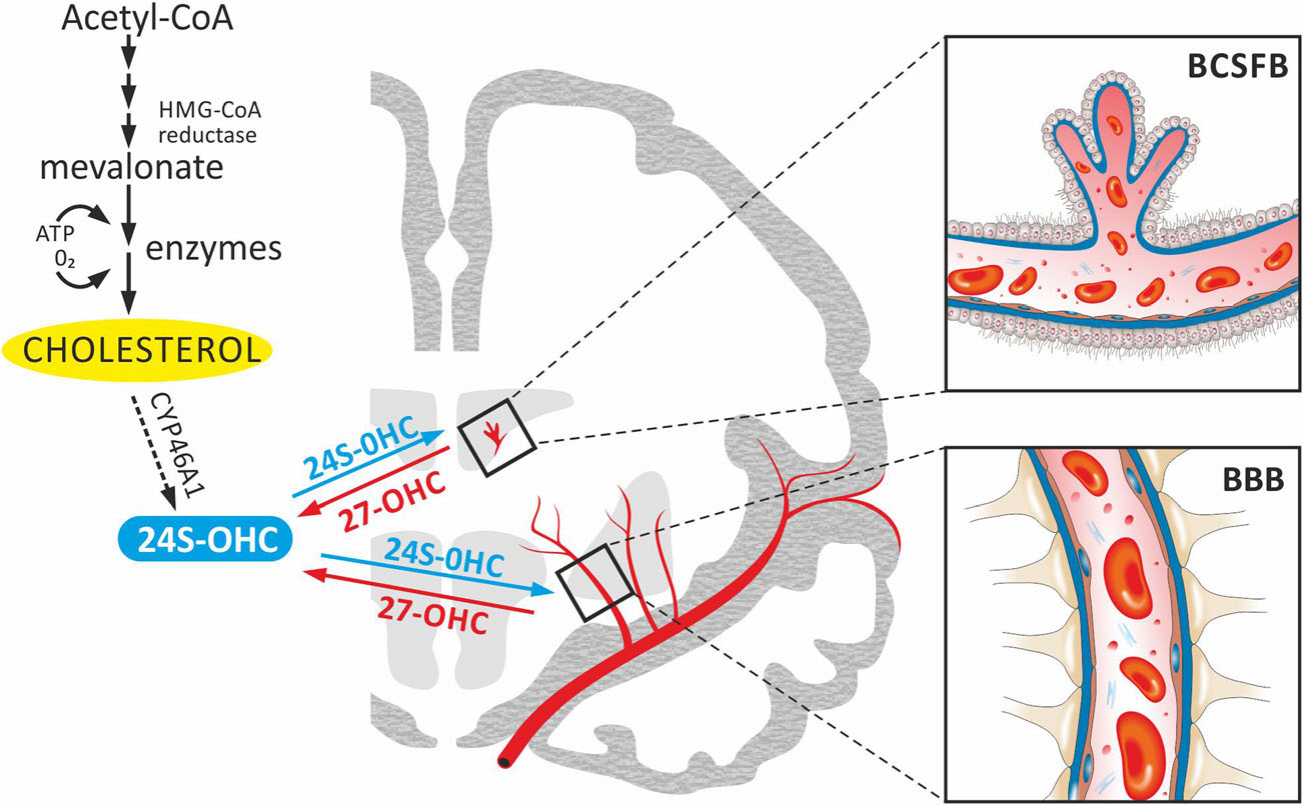
Cholesterol synthesis and metabolism in the brain. Brain cholesterol is synthesized from acetyl-CoA to mevalonate by HMG-CoA reductase. Subsequently, it is converted to cholesterol in numerous enzymatic reactions, dependent on access to ATP and O_2_. Excess cholesterol is converted to the 24S-OHC and removed from the brain because its accumulation is highly toxic to neurons and may induce their apoptosis. A part of the cholesterol pool is taken up from the general pool as the 27-OHC by diffusion. This is possible because of the oxysterols’ ability of crossing the functional barrier systems: BBB (formed by cerebrovascular endothelial cells) and BCSFB (formed by epithelial cells of the choroid plexus). Abbreviations: *24S–OHC* 24(S)-hydroxycholesterol, *27-OHC* 27-hydroxycholesterol, *acetyl-CoA* acetyl coenzyme A, *BBB* the blood brain barrier, *BCSFB* the blood–cerebrospinal fluid barrier, *HMG-CoA reductase* 3-hydroxy-3-methylglutaryl-CoA reductase

In the brain cholesterol is synthesized in situ mainly by astrocytes and oligodendrocytes (Liu et al. 2010a). In essence, it is synthesized from acetyl coenzyme A (acetyl-CoA) in a series of reactions, catalysed by over 20 enzymes, requiring the considerable energy in the form of 3 adenosine triphosphate molecules (ATP), and oxygen supply (Fig. 1). Critical in this process is the conversion of acetyl-CoA to mevalonate by 3-hydroxy-3-methylglutaryl-CoA reductase (HMG-CoA reductase), as the rate-limiting and irreversible step in cholesterol synthesis (Gaylor 2002).

Cholesterol pool in the CNS is almost completely isolated from the blood circulation by the functional barrier systems: the BBB, formed by cerebrovascular endothelial cells and the blood–cerebrospinal fluid barrier (BCSFB), formed by epithelial cells of the choroid plexus (Zlokovic 2008; Lehtinen et al. 2013). However, part of the cholesterol pool is exchanged between the brain and the blood circulation in the form of oxysterol metabolites, such as 27-hydroxycholesterol (27-OHC) and 24S–hydroxycholesterol (24S–OHC) by diffusion (about 6–7 mg of 24S–OHC and 5 mg of 27-OHC per day) (Björkhem 2006; Gosselet et al. 2014). A small amount of cholesterol can be taken up from the general pool as 27-OHC or via the scavenger receptor class B type I (SR-BI). The 27-OHC is a metabolite of cholesterol synthetized in various cells of the body, in the reaction catalysed by the sterol 27-hydroxylase (CYP27A1). Capable of crossing the BBB and BCSFB, it can supply the cholesterol pool in the brain, entering from the circulatory system (Kim et al. 2007). The SR-BI, located in the brain capillary endothelial cells, is responsible for selective lipid uptake, including cholesterol from the high-density lipoprotein (HDL) and the low-density lipoprotein (LDL) (Balazs et al. 2004). Another important oxysterol that also can cross both barrier systems is 24S–OHC, which is produced in the brain by the enzymatic reaction catalysed by a specific cholesterol 24-hydroxylase (CYP46A1), expressed in neurons but not in astrocytes (Liao et al. 2011). The 24S–OHC has been also found in the retina, where it diffuses freely thorough the cell membranes into the vascular system (Liao et al. 2011).

The rate of cholesterol metabolism in the brain (and other body organs) can be determined by calculating the ratio of 24S–OHC to 27-OHC measured in the blood plasma. It is assumed that one of the causes of the higher levels of 24S–OHC in the plasma is cerebrovascular disfunction during the early stages of neurodegenerative process, which could be the result of greater permeability of the BBB (Hughes et al. 2012). Increased level of 24S–OHC has been detected in the plasma of patients with vascular dementia and recently diagnosed with AD. There has also been observed a correlation between higher 24S–OHC concentration in plasma and development of cognitive impairment in AD patients (Lütjohann et al. 2000). However, in the case of long-term and advanced stage of AD, the level of 24S–OHC in the plasma has been reduced (Papassotiropoulos et al. 2000).

#### Astrocytic contribution to cholesterol synthesis increases during brain development

Due to the maximum intensity of myelination, cholesterol synthesis in astrocytes and neurons reveals the highest level during embryogenesis and childhood (Dietschy and Turley 2004). Then, completely differentiated neurons decrease their capacity for the cholesterol biosynthesis. At this stage, they are less efficient in compensation for the cholesterol deficiency than astrocytes. Consequently, they become dependent on astrocytes, as the largest producers and suppliers of cholesterol in the adult brain (Nieweg et al. 2009).

**Fig. 2:**
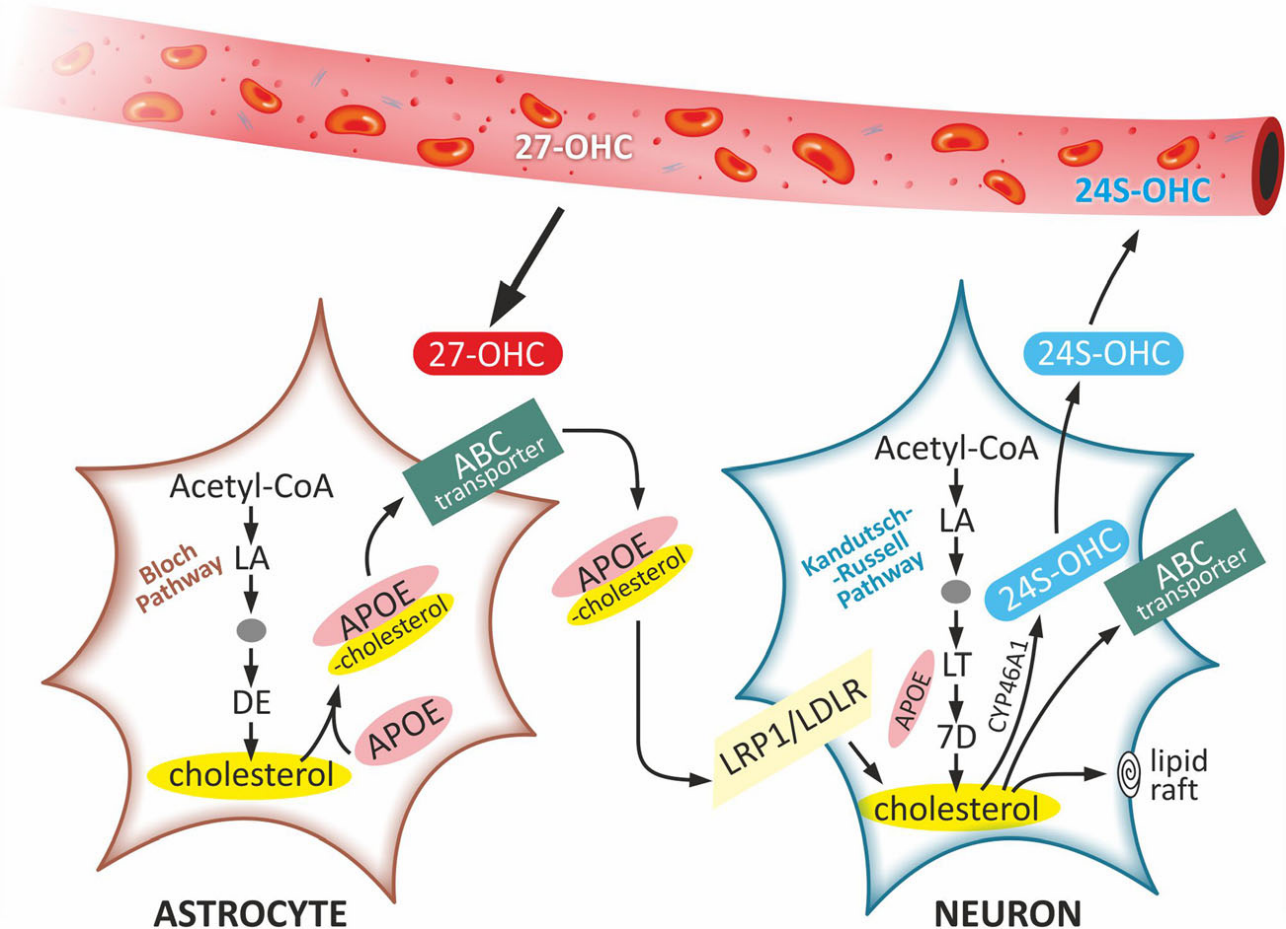
Cholesterol synthesis and metabolism in astrocytes and neurons. Cholesterol is biosynthesized in astrocytes via Bloch pathway, while in neurons via Kandutsch-Russell pathway. In both pathways, Acetyl-CoA is converted to LA and definitively transformed to cholesterol, but they differ in level of the cholesterol precursors: while DE is the main precursor in astrocytes, as LA, LT and 7D are present mainly in neurons. Synthesized in astrocytes APOE is secreted in the form of APOE-containing cholesterol by the ABC transporter and absorbed by neurons via the LRP1/LDLR receptors by endocytosis. The excess cholesterol is converted into 24S–OHC by Cyp46A1 in order to excrete it across BBB and BCSFB to the circulation. It can also be released by the ABC transporter or utilized for the formation of new cellular membranes and microdomains as the lipid rafts. Alternatively, small amount of cholesterol can also be taken up as 27-OHC from the circulation into the brain. Abbreviations: *7D* 7-dehydrocholesterol, *24S–OHC* 24(S)-hydroxycholesterol, *27-OHC* 27-hydroxycholesterol, *Acetyl-CoA* acetyl coenzyme A, *APOE* apolipoprotein E, *ABC transporter* ATP-binding cassette transporter, *Cyp46A1* cholesterol 24-hydroxylase, *DE* desmosterol, *LA* lanosterol, *LT* lathosterol, *LRP1/LDLR receptors* low-density lipoprotein receptor-related protein 1/ low-density lipoprotein receptor

There are two main pathways of cholesterol biosynthesis in the brain: the Bloch pathway in the astrocytes and the Kandutsch-Russell pathway in the neurons (Fig. 2) (Nieweg et al. 2009). It is worth mentioning that astrocytes and neurons reveal different concentrations of the cholesterol precursors. Whereas desmosterol (DE) is the main cholesterol precursor in the astrocytes, lanosterol (LA), lathosterol (LT) and 7-dehydrocholesterol (7D) prevail in the neuronal cells (Nieweg et al. 2009). Hence, the synthesis of cholesterol in both cellular populations must rely on utilization of different precursors predominating in each of them. However, the totally developed neurons exhibit very low levels of enzymes involved in the intermediate reactions of the lanosterol such as 24-dehydrocholesterol reductase (DHCR24) and lanosterol 14-alpha demethylase (CYP51). This indicates ineffective character of the lanosterol conversion into cholesterol in the neurons (Nieweg et al. 2009). Furthermore, several studies suggest that cholesterol synthesis in neurons occurs only in the soma and the final product must be transported to the axons and dendrites (Vance et al. 1994).

The observations carried out by Oberheim et al. on the comparative material of human and mouse tissues, highlighted differences in the size and complexity of astrocytic cells - mice astrocytes are smaller and less complex than astrocytes in human tissues (Oberheim et al. 2009). Transcriptomic studies of human and mouse astrocytes have shown that more than 600 human astrocyte-enriched genes have not been localized in murine models (Zhang et al. 2016). Interestingly, transplantation of human glial progenitor cells is associated with improved learning and memory (Han et al. 2013). It is noteworthy that the phenotypic differences associated with astrocytes of human and mouse tissue can have a major impact on cholesterol synthesis and metabolism in the brain.

Metabolic cooperation between the astrocytes and neurons in the cholesterol synthesis depends on their energetic status. Cholesterol synthesis requires a lot of energy, which under physiological conditions is supplemented by astrocytes during brain development (Nieweg et al. 2009). Due to this cooperation neurons save energy, which can be used for maintenance of the membrane resting potential, generation of the electrical activity and synaptic plasticity. Although this relationship is beneficial for the both cell types in physiological conditions, it is unknown which of them mostly complement deficiency in the cholesterol synthesis in pathological processes.

### Maintaining of the brain cholesterol homeostasis requires precisely regulated functional cooperation between astrocytes and neurons

The cholesterol homeostasis is essential for maintaining of the brain structure and function. Disturbances of the synthesis, as well as its abnormal efflux or influx, can result in a deficiency or a significant accumulation of the cholesterol in the brain. Since the astrocytes and neurons are crucial for cholesterol metabolism, its synthesis and transport between these cells are precisely regulated by the modulatory factors.

#### Several regulatory mechanisms of the astrocytic and neuronal metabolism control the cholesterol homeostasis in physiological conditions

Despite the fact that astrocytes are the main producers of cholesterol in the adult brain, the maintenance of its metabolic homeostasis is equally controlled by astrocytes and neurons (Brown and Goldstein 1999). Both these cell types have developed several mechanisms regulating cholesterol homeostasis. One of them is a negative feedback regulatory system, enhancing the synthesis, excretion and transport of cholesterol under physiological conditions to maintain its optimal level in the cells. This mechanism is based on the sterol regulatory element-binding protein 2 (SREBP2) associated with the membranes of the endoplasmic reticulum (ER), which regulates the transcription of genes encoding enzymes involved in cholesterol synthesis (Brown and Goldstein 1999). Several studies have shown that deficiency of cholesterol or enzymes involved in its synthesis, as well as decreased cholesterol transport and uptake by neurons, evoke SREBPs-induced enhancement of genes transcription, which encode cholesterol biosynthetic enzymes in the astrocytes (Brown and Goldstein 1999).

On the contrary, when the cholesterol level exceeds the physiological norm, or the level of enzymes involved in its synthesis is too high, the excess of cholesterol is converted to the 24S–OHC by Cyp46A1 and eliminated from the brain in the form of HDL, since 24S–OHC accumulation is highly toxic to neurons and may induce their apoptosis (Matsuda et al. 2013). The expression of Cyp46A1 decreases in the neurons and accordingly increases in the astrocytes of patients with advanced AD (Matsuda et al. 2013). In addition, the 24S–OHC is an activator of nuclear transcription factors (e.g. liver X receptors (LXRs)), which increase the expression of genes encoding ATP-binding cassette transporters (e.g. ABCA1, ABCG1 and ABCG4), involved in cholesterol transport across the astrocytic and neuronal membranes. It also increases the production of apolipoprotein E (APOE), which is the main lipid carrier protein in the astrocytes (Abildayeva et al. 2006).

The level of the 24S–OHC in blood may be used as a sensitive marker of pathological processes and changes in the cholesterol homeostasis in the brain (Bogdanovic et al. 2001). The 27-OHC and 24S–OHC are natural inhibitors of cholesterol synthesis in vitro and play an important role in neurodegenerative diseases (Saeed et al. 2014). The study showed evidence that 24S–OHC and 27-OHC serve as inhibitors of the amyloid β (Aβ) peptide formation, whereas 27-OHC had significantly less ability to inhibit this reaction (Brown et al. 2004). The increased permeability of the BBB may lead to an augmented influx of the 27-OHC to the brain and efflux of the 24S–OHC. The pathway for cholesterol elimination has an important regulatory function due to the influx of 27-OHC to the brain (Kim et al. 2007). It is also possible that cholesterol can penetrate the neuronal cell membrane using the “flip-flop” mechanism, however controlling the cholesterol level with this type of transport should be further investigated (Róg et al. 2008).

### Modulators of cholesterol transport in the brain contribute to its homeostasis in physiological and pathological conditions

Both neurons and astrocytes synthesize cholesterol in situ*,* but differ considerably in terms of its metabolism, in particular, regarding the mechanisms of its synthesis, maintaining of its homeostasis, as well as the brain cholesterol efflux and influx (Björkhem et al. 2010). Single peptide modulators, as well as complex systems of receptors are involved in regulation of cholesterol transport. The first group includes apolipoprotein E and ATP-binding cassette transporter, while the second includes liver X receptors and LDL receptors. The coordinated cooperation between them ensures the maintenance of cholesterol homeostasis in the brain.

#### Apolipoprotein E

Apolipoprotein E (APOE) is a lipid carrier of cholesterol, involved in its transport in both physiological and pathological conditions (Mahley and Rall 2000). APOE is a 299 amino acid apolipoprotein, encoded by the *APOE* gene, which is highly expressed in many human organs particularly on the liver and the brain (Mahley and Rall 2000). In physiological state APOE is highly expressed in the astrocytes, but not in the neurons. However, in the brain pathological processes such as excitotoxic injury, the neurones can also express APOE (Xu et al. 1999). The astrocytes synthesize APOE and secret it to the extracellular space in the form of APOE-containing lipoproteins and phospholipids, that transport cholesterol, which is then absorbed by neurons during receptor-mediated endocytosis (Fig. 2). The cholesterol taken up by the neurons can be further converted into the 24S–OHC, in order to eliminate it from the brain (Hayashi et al. 2004). Alternatively, it can be utilized for the formation of new cellular membranes and microdomains in the form of lipid rafts (Lahiri 2004).

The APOE is stable only when it is bound to the lipids, whereas in the unbound state it is degraded and its level drastically decreases (Hirsch-Reinshagen et al. 2004). It has been previously reported that APOE level is reduced in *abca1*-gene-knockout mice (Wahrle et al. 2008). The product of this gene is necessary for the APOE lipidation. Studies have shown that mice with a reduced level of APOE do not display significant changes in cholesterol levels, whereas in mice lacking APOE there is observed a reduction in both cholesterol and its precursors compared to control animals (Levi et al. 2005; Jansen et al. 2009). It is not clear whether changes in the cholesterol level result directly from the APOE deficit. Interestingly, a reduced level of APOE does not influence the cholesterol homeostasis, which could indicate an existence of alternative regulatory mechanism (Anderson et al. 1998). Studies showed that the APOE deficit during brain development coincided with abnormal synaptic and dendritic densities in the hippocampus, but without significant functional consequences. In the adult mice, however, it has been accompanied by serious behavioural changes and neurological symptoms (Anderson et al. 1998). A relationship between the level of the APOE in brain and development of AD has been suggested by some authors (Corder et al. 1993; Castellano et al. 2011). However, the exact mechanism remains unknown.

In the human brain APOE is expressed in three isoforms: ApoE2, ApoE3 and ApoE4 (Corder et al. 1993). Whereas in the mouse brain, ApoE3 and ApoE4 are expressed by astrocytes both during development and in adult mice (Sun et al. 1998). It seems intriguing that the cellular source of APOE can have a decisive influence on regulating neurite overgrowth. The results obtained by the Holtzmans group show that apolipoproteins containing ApoE3 have a different neurobiological activity than ApoE4-containing lipoproteins. It is evident that hippocampal neurons are defined by increased neurite growth in the presence of ApoE3-secreting astrocytes compared to ApoE4-secreting astrocytes or ApoE knock-out astrocytes in primary cultures (Sun et al. 1998).

Recent studies show the role of the APOE genotype in modulating the astrocyte phagocytic capacity and in controlling the rate of synapse pruning and turnover by astrocytes in vitro and in vivo (Chung et al. 2016). ApoE2 allel increases the rate of synaptic phagocytosis by astrocytes, while Apo4 prevents it, which in consequence leads to excessive accumulation of senescent synapses. Moreover, recent research shows that the APOE allele also affect the amount of C1q protein accumulated in the hippocampus, which may be the determinant of the accumulation of senescent synapses (Chung et al. 2016). ApoE2 knock-in animals are characterized by a reduction in C1q protein accumulation, while in ApoE4 animals the level is significantly increased relative to ApoE3 animals. Therefore, the regulation of the phagocytosis rate, conditioned by APOE isoforms, occurs by modulating one or more phagocytic pathways (Chung et al. 2016).

Corder et al. showed that presence of ApoE2 was related to reduced risk of AD (Corder et al. 1993). The ApoE4 increases the risk of AD and its presence correlates with the lower age of its onset. This protective relationship between ApoE4 and AD may be related to the partially defective phagocytic capacity of astrocytes (Chung et al. 2016). Interestingly, there is evidence that APOE binds to Aβ and plays role in β-amyloid plaques formation (Vance and Hayashi 2010). In addition, various APOE isoforms have different capacity for binding with Aβ, which seems to be related with different lipidation capacity. The APOE2 and APOE3 isoforms become lipidated more easily than ApoE4, so it seems that the APOE lipidation can be an important process involved in AD (Castellano et al. 2011).

#### ATP-binding cassette (ABC) transporters

ATP-binding cassette (ABC) transporters are key regulators of lipid homeostasis in the brain (Hirsch-Reinshagen et al. 2004). The ABC transporters, that regulate cholesterol homeostasis, belong to two families: ABCA and ABCG. They are responsible for lipid transport across the cell membrane (Oram and Heinecke 2005). Both the astrocytes and the neurons express: ABCA1, ABCG1 and ABCG4 transporters. The ABCA1 transporter has a broad substrate specificity which enables it to transport cholesterol and phospholipids (Oram and Heinecke 2005). Both ABCA1 and ABCG1 are involved in the cholesterol efflux from astrocytes (Fig. 2). Whereas silencing or increasing expression of ABCA1 and ABCG1 has no effect on the cholesterol efflux from the neurons, it reduces and increases cholesterol efflux from the glial cells, respectively.

ABCA1 is an important factor maintaining the proper level of APOE and thus cholesterol homeostasis in the brain (Hirsch-Reinshagen et al. 2004). Deficiency in this transporter may result in poor lipidation and degradation of APOE and significant reduction of cholesterol levels (Hirsch-Reinshagen et al. 2004). In brain, the low expression of ABCA1 increases accumulation of Aβ, whereas its high expression reduces the amyloid deposition in the murine model brain of AD (Wahrle et al. 2005, 2008). The levels of ABCA1, ABCG1 and APOE may be increased by the 24S–OHC of neuronal origin, which indirectly regulates the cholesterol efflux from the glial cells (Abildayeva et al. 2006).

Kim et al. showed that the expression of ABCG4 was much higher in neurons than in glial cells (Kim et al. 2006). Consequently, ABCG4 is involved in the cholesterol efflux from neurons, but not from astrocytes (Chen et al. 2013). Thus, the increase in expression of this transporter activates the efflux of cholesterol only from the neurons, not from glial cells.

#### LDL receptors

There are two functionally important LDL receptors: the prototypic low-density lipoprotein receptor (LDLR) and the low-density lipoprotein receptor-related protein 1 (LRP1) that are involved in the cholesterol transport in the human brain (Fig. 2) (Herz 2009). Although both of them are expressed in astrocytes and neurons, the LDLR is highly expressed in the astrocytes, whereas LRP1 is expressed mainly in the neurons (William Rebeck et al. 1993). The LDL receptors play an important role in lipid homeostasis and intracellular signalling (Pfrieger and Ungerer 2011). They bind different ligands, including apoE-containing lipoproteins, and regulate the integrity of the BBB, what makes them important factors regulating cholesterol efflux from the cells (Strickland et al. 2014). Liu et al. showed increased level of APOE in LDL-receptor knockout mice, which resulted in decrease of the cholesterol levels in the brain (Liu et al. 2010b). Due to the involvement of the LDL receptors in the clearance of Aβ associated with BBB permeability, it was suggested that these modulating agents could be potentially considered for treatment of AD (Martiskainen et al. 2013).

#### Liver X receptors

Liver X type α and β receptors (LXRs) belong to a family of nuclear receptors involved in regulation of cholesterol metabolism. Furthermore, stimulation of LXRs is responsible for astrocytic recruitment to the inflammatory response, in the course of pathological processes (Edwards et al. 2002). Previous studies have shown that LXRs inhibit cholesterol synthesis, induce expression of the ABC transporters and enhance the APOE lipidation (Abildayeva et al. 2006). Consequently, in vitro studies show that the cholesterol efflux is stimulated, which results in the exhaustion of cholesterol pool in the astrocytic culture. In addition, LXRs activity in the astrocytes can be regulated by the level of 24S–OHC released by the neurons, which augments the effect of cholesterol efflux (Abildayeva et al. 2006).

Deficiency of either LXRα or LXRβ in the APP/PS1 transgenic mouse model of AD increases the deposition of Aβ in the brain, through negative regulation of microglial phagocytosis, resulting in dramatic increase in lipid accumulation and development of neurodegenerative disorders (Zelcer et al. 2007). The LXRs inhibit the processing of amyloid precursor protein (APP) by modulating membrane cholesterol levels via ABCA transporters and accelerate Aβ clearance by inducing APOE lipoprotein secretion (Riddell et al. 2007). Due to the ability to modulate metabolic factors involved in the pathogenesis of AD and to regulate the inflammatory response by inhibiting glial cells activity, the LXRs seem to be an attractive therapeutic target for neurodegenerative diseases (Zelcer et al. 2007).

## Brain cholesterol metabolism in pathological conditions

### High-cholesterol diet contributes to dysregulation of brain cholesterol homeostasis and development of neurodegenerative diseases

The human brain requires a sufficient level of cholesterol during its development and proper functioning later in life. However, a long-term high-cholesterol diets contribute to the increase of plasma cholesterol and, most importantly, disturb the cholesterol brain homeostasis (Vance 2006). This initiates pathological processes, such as accumulation of Aβ, hyperphosphorylation of Tau protein and neuronal death (Vance 2006). Previous studies have indicated a coexistence of hypercholesterolemia with pathological signs of AD, but the involved mechanisms are still unclear (Ehehalt et al. 2003).

#### Astrocytic activation is initiated in the early stages of hypercholesterolemia

Chen et al. showed that the long-term high-cholesterol diet triggered astrocytic activation in the murine hippocampus (Chen et al. 2016). This led to increase in expression of proteins involved in the cholesterol transport across cell membranes such as APOE and aquaporin 4 (AQP4), as well as increase in expression of proinflammatory cytokine IL-1β (Chen et al. 2016). High expression of APOE during the early stages of hypercholesterolemia also has an impact on the homeostasis of cholesterol in the brain (Chen et al. 2016).

The increased expression of AQP4 in mice on the high-cholesterol diet is due to the greater demand for cholesterol transport, as well as the need to remove the soluble Aβ (Xiao and Hu 2014). This may suggest an important role of AQP4 in defence mechanisms that are initiated by the high cholesterol levels. Therefore, mice with AQP4 deletion show increased levels of Aβ accumulated in the brain. Xu et al. hypothesize that AQP4 plays a role in the Aβ transport and influences its metabolism in order to regulate cholesterol homeostasis in the hypercholesterolemia (Xu et al. 2015).

In vivo studies in mouse model showed increased expression of IL-1β in the reactive astrocytes during the early stages of AD (Apelt and Schliebs 2001). The similarity of immunological reactions observed in the hypercholesterolemia and the early stages of AD, suggests the crucial role of astrocytes in both these processes. It is possible that sustained overexpression of IL-1β reduces the burden of Aβ in the brain of mice with AD (Ghosh et al. 2013). On the other hand, release of inflammatory factors promotes Aβ accumulation, that leads to changes in oxidative status and neurodegeneration (Chen et al. 2016).

In summary, these results suggest that the cholesterol contained in the diet is involved in the pathogenesis of AD and can modulate the reaction of astrocytes, but initially does not cause significant functional changes in the neurons.

#### Hypercholesterolemia contributes to development of neurodegenerative changes characteristic for Alzheimer disease

The increased cholesterol concentration in the brain have been claimed to accelerate the development of AD, whereas its reduced level supports a beneficial therapeutic effect (Ehehalt et al. 2003). The long-term high cholesterol diet, that induces hypercholesterolemia, increases generation and accumulation of Aβ in the brain of transgenic mice. This leads to increased expression and hyperphosphorylation of Tau protein (Ghribi et al. 2006). However, these hypercholesterolemia-related changes are time-dependent and indirectly influence each other (Chen et al. 2016).

Chen et al. showed that mice on the long-term high-cholesterol diet had an increased expression of presenilin 1 (PS1) and insulin-degrading enzyme (IDE), which are involved in degradation of Aβ secreted into the extracellular space (Chen et al. 2016). This might be a mechanism regulating the formation and accumulation of proteins associated with AD pathology and maintaining cholesterol homeostasis in the brain. However, the direct involvement of astrocytes and neurons in these processes remains unclear, due to the fact that the study has been conducted on the homogenate from murine hippocampus.

Whereas AQP4 deletion mice show increased levels of Aβ accumulated in the brain, it is likely that AQP4 also takes part in the Aβ transport and maintaining the redox homeostasis in the glial cells of mice with hypercholesterolemia (Xu et al. 2015). High expression of APOE during the early stages of hypercholesterolemia also has an impact on the cholesterol homeostasis in the brain. On the other hand, other studies show that the release of inflammatory factors in the brain promotes Aβ accumulation, leading to changes in the oxidative status and the development of neurodegeneration (Chen et al. 2016). These data indicate a close correlation between the high-cholesterol diet resulting in the hypercholesterolemia and pathogenesis of AD. Monitoring of the cholesterol levels in the AD patients is significant importance for the assessment of the AD progress.

One of the major subtypes of dyslipidemia is familial hypercholesterolemia, which is an autosomal genetic disorder that is directly or indirectly associated with LDL receptor dysfunction, leading to defective catabolism of LDL and long-term hypercholesterolemia (de Oliveira et al. 2014). This condition is defined by defective catabolism of LDL, leading to an increase in total plasma cholesterol level and long-term hypercholesterolemia. An increasing number of clinical evidence points to the fact that middle-aged patients with familial hypercholesterolemia showed a high incidence of mild cognitive impairment, which in most cases results in AD development (Zambón et al. 2010). Mouse models of familial hypercholesterolemia also defined oxidative stress and increased BBB permeability, and most importantly, increased susceptibility to Aβ-induced neurotoxicity (de Oliveira et al. 2014). There have also been behavioural changes in the form of memory deficits and spatial learning. These studies provide new evidence for the association of familial hypercholesterolemia with pathological changes associated with AD, which opens new possibilities for investigating the relationship between lipid metabolism and neurodegenerative diseases.

### Oxidative stress contributes to development of neurodegenerative changes by influencing of the cholesterol metabolic balance in the brain

Studies of various pathological processes in the brain showed that oxidative stress is a process present in the majority of them. Consequences of the oxidative stress vary depending on the studied pathological process and relate to various aspects of metabolism in neurons and glia.

#### Astrocytes play both detrimental and beneficial role in the course of the oxidative stress

Growing body of evidence indicates critical contribution of astrocytes to the oxidative stress in the brain. It should be emphasized that the role of astrocytes in the course of oxidative stress is both negative and positive. Oxidative stress causes disruption of the glucose energy transformation and ATP production in brain, sodium and potassium ionic imbalance, impaired uptake of defined neurotransmitters, release of the inflammatory response mediators and initiation of the cell death (Torp et al. 1994; Mashayekhi et al. 2010; Nagayasu et al. 2014).

**Fig. 3:**
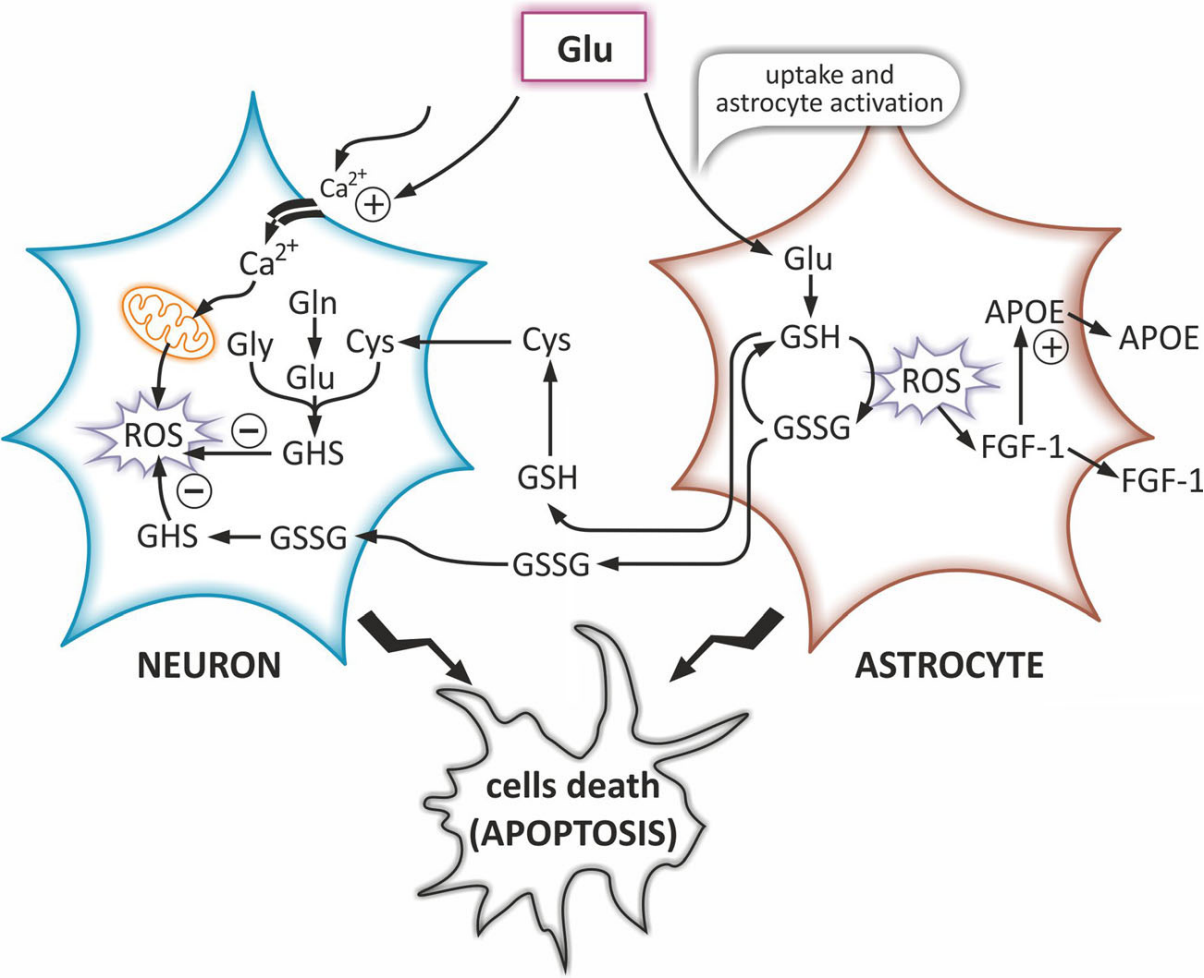
Impact of oxidative stress on cholesterol homeostasis in astrocytes and neurons. Glutamatergic neurotransmission is associated with the influx of Ca^2+^ ions inside the neuron that in turn induces the ROS production in the mitochondria. This can lead to neuronal damage and consequently triggers antioxidant systems that increase the production and secretion of the GSH. Astrocytes uptake Glu and thus remove it from the synaptic cleft in order to protect neurons from the excitotoxic effect. Moreover, Glu is one of the substrates for GSH synthesis in astrocytes. Under the oxidative stress conditions, there are also produced ROS in astrocytes, which contribute to GSH oxidation into GSSG. Both the GSH and the GSSG are released into the extracellular space and converted by the ectoenzymes to precursors of GSH synthesis in neurons. In the presence of ROS, FGF-1 enhances the synthesis of APOE and both the FGF-1 and the APOE are released from astrocytes. Prolonged exposure of astrocytes and neurons to oxidative stress leads to initiation of cell death processes. Abbreviations: *APOE* apolipoprotein E, *Glu* glutamate, *GSH* glutathione, *GSSG* glutathione disulfide, *FGF-1* a fibroblast growth factor 1, *ROS* reactive oxygen species

However, despite the above-mentioned glia-related detrimental effects of oxidative stress on the CNS, one of the most important astrocytic function is the protection of neurons from the oxidative stress-induced neurodegenerative changes (Torp et al. 1994). To the most important factors limiting the effects of the oxidative stress in the brain belong: an increased production and secretion of glutathione (GSH) and glutathione disulfide (GSSG) (Torp et al. 1994; Hirrlinger et al. 2001). The GSH is the major antioxidant involved in the detoxification of ROS, and thus in the neuronal protection and regulation of redox homeostasis (Dringen 2000). In the human brain, much more of the GSH is released from astrocytes than from neurons, but the release from both of them can be significantly increased by oxidative stress (Sagara et al. 1996; Hohnholt and Dringen 2014). Excitotoxic glutamate (Glu) is one of the substrates of GSH synthesis and the concentration of GSH is regulated by a negative feedback mechanism (Fig. 3). To prevent the excitotoxic raise of the extracellular Glu, astrocytes not only increase its uptake but also increase the GSH production. The GSH is released into the extracellular space and converted by the ectoenzyme ɣ-glutamyl transpeptidase (ɣGT) to precursors of GSH synthesis in neurons.

Oxidative stress also causes the release of GSSG from the astrocytes (Hirrlinger et al. 2001). The GSSG is the product of the nonenzymatic reactions of GSH with ROS (Hirrlinger et al. 2001). In physiological conditions, astrocytes do not release GSSG (Hirrlinger et al. 2001). Hence, measurement of the extracellular GSSG can be an indicator of the oxidative stress (Sucher and Lipton 1991). Moreover, GSSG is a modulator of Glu receptors, important for protection of neurons against Glu excitotoxicity (Sucher and Lipton 1991). Along with many other factors affecting the oxidative stress, the oxidation processes in the cells can also be accelerated by the high-cholesterol diet and hypercholesterolemia (Crawford et al. 2012).

Another factor potentially involved in oxidative stress in the astrocytes is a fibroblast growth factor 1 (FGF-1). In long-term astrocytic culture, where oxidative stress was triggered by H_2_O_2_, FGF-1 enhanced the synthesis and release of APOE in order to up-regulate APOE/HDL generation. Consequently, this increased the cholesterol synthesis in vitro (Nagayasu et al. 2014). The FGF-1 is expressed in reactive astrocytes in the course of AD (Mashayekhi et al. 2010). Clinical studies showed that in AD patients the FGF-1 level was higher in the cerebrospinal fluid (CSF) and serum compared to the control group. Due to the fact that oxidative stress is an important process in the pathogenesis of AD, one can hypothesize that increased level of the FGF-1 is associated with the presence of ROS and the detrimental mechanisms in which they are involved (Mashayekhi et al. 2010). It can also be caused by gliosis resulting from brain damage occurring the course of neurodegenerative diseases (Mashayekhi et al. 2010). Future studies should focus on elucidation of the mechanisms involved in the release of the FGF-1 under the influence of ROS. The key role in these mechanisms may play the FGF-1-induced secretion of the APOE, that potentially may influence cholesterol metabolic homeostasis under the oxidative stress conditions.

The relationship between oxidative stress and hypercholesterolemia has been demonstrated in preclinical studies. Few studies showed possible mechanisms of this relationship and metabolites that might be involved (Aytan et al. 2008). The current state of knowledge does not allow to clearly define pathways modulation of which could protect the brain cells from oxidative stress during hypercholesterolemia.

#### Impact of the oxidative stress on AD progression is mainly related to impairment of the astrocytic function

Increasing evidence indicates that oxidative stress plays an important role in the pathogenesis of the AD. Oxidative damage in the brain of patients with AD is associated with both a reduced level of the GSH in the cells and changes in the concentration and activity of the antioxidant enzymes (Zhang et al. 2012). However, further studies are required to explain this process. In the course of AD, the release of GSH from astrocytes raises without increase in its synthesis. Hence, the total GSH level decreases with time (Zhang et al. 2012). Furthermore, the monomeric Aβ deposited in the brain leads to increase in the expression of the ABCC1 transporter, which is the main pathway of GSH releasing by ATP-dependent mechanism of substrate transport (Ye et al. 2015). The monomeric Aβ also stimulates Cx43 hemichannels involved in the release of GSH and excitotoxic Glu from astrocytes to the extracellular space in response to increased level of intracellular Ca^2+^ ions (Ye et al. 2015).

In summary, it is possible that astrocytes have developed a GSH release mechanism into the extracellular space to provide temporary protection from oxidative stress and to supply precursors of GSH synthesis in the neurons. Disturbances of GSH release from the astrocytes and deficiency of GSH synthesis in the neurons lead to changes in their oxidative status. The consequence of those changes may be development of the oxidative stress, which promotes the age-dependent neurodegeneration.

### Impairment of the brain cholesterol homeostasis contributes to development of the angiopathic changes related to neurodegenerative and metabolic diseases

The cholesterol is directly or indirectly involved in numerous metabolic pathways and the protective mechanisms in the brain. Disturbances in cholesterol synthesis and metabolism can cause both cerebrovascular and neurodegenerative diseases. The concept of the neurovascular unit, assuming close cooperation between three components of the nervous tissue (i.e. neurons, blood vessels and neuroglia), proved to be useful for understanding and explaining the pathogenesis of many neurological diseases (Zlokovic 2011). The damage to neuronal component is associated with the impaired neuronal excitability and synaptic plasticity, which may consequently lead to the cognitive or memory deficits. Dysfunction of the neuroglial cells, in particular astrocytes, leads to impaired energy metabolism, as well as disturbances in the metabolic homeostasis of the nervous tissue (Torp et al. 1994; Mashayekhi et al. 2010; Nagayasu et al. 2014). Finally, damage to the cerebrovascular component of the NVU results in the increased permeability of the BBB, pathological re-building of the vascular wall in the course of angiopathic process and ultimately, in dysregulation of the cerebral blood flow (CBF). The pathogenesis of most brain diseases involves simultaneous damage to all three components of the neurovascular unit. Consequently, the effects of cholesterol metabolism disorders must be assessed in relation to all NVU elements.

#### Amyloid angiopathy in the course of AD disintegrates neurovascular coupling and deteriorates mechanisms of CBF regulation

The accumulation of Aβ in the blood vessel walls, that leads to cerebral amyloid angiopathy (CAA), is one of the most important processes in the pathogenesis of AD. The CAA development is related to an impaired removal of Aβ from the perivascular space (Shin et al. 2007). The consequence of the CAA is inhibition of angiogenesis and the increasing weakness of the cerebral vessel walls, which results in disruption of the vascular tone and deterioration of the CBF (Shin et al. 2007). The accumulation of Aβ and the development of CAA are most frequently observed in leptomeningeal and large penetrating cortical vessels of the cerebral cortex (Herzig et al. 2004). The parenchymal amyloid is removed via bulk flow drainage along the basement membrane of the vessel (Weller et al. 2008). CAA in AD patients leads to reduced Aβ clearance from the brain parenchyma, which clearly indicates their close association.

Taking into account that the neurovascular unit is involved in the clearance of Aβ, any changes in its structure reduce the efficiency of the Aβ removal and cause its accumulation in the brain (Zlokovic 2011). However, Aβ secreted into the cerebral interstitial fluid is removed from the brain by bulk flow along the basement membranes of capillary and artery walls (Hawkes et al. 2012). Under physiological conditions, matrix metalloproteinase-9 (MMP-9) is involved in the maintenance of the NVU integrity. Additionally, the MMP-9 degrades Aβ fibrils and in this way contributes to the removal of amyloid plaques from the human brain (Yan et al. 2006). The excessive activation of the MMP-9 in the brain, however, may cause the NVU disorders and leads to disturbances in Aβ clearance that induces neurodegeneration. Clinical studies showed increased levels of the MMP-9 in the neurons but not in the microvessels in the brain of patients with AD (Asahina et al. 2001; Thirumangalakudi et al. 2006).

Recent evidence supports the hypothesis that both AQP4 and Aβ can have an effect on the brain-wide pathway for fluid transport in the mouse brain, known as glymaphatic drainage pathways (Iliff et al. 2012). Mice lacking of AQP4 in astrocytes showed slowed influx of CSF as compared to control mice, which resulted in a decrease of ~70% of interstitial solute clearance (Yao et al. 2008). Mice with an incorrectly localized form of AQP4 or completely deprived of AQP4 in astrocytes were characterized by swollen endfeet and neuropathological changes within the arteries. The dimension of glymaphatic drainage pathways in the interstitial brain solutes clearance, probably also a soluble Aβ peptide, may be prospective in research on the development of AD in patients (Iliff et al. 2012). It has been revealed that abnormalities in the AQP4 functioning or AQP4 gene deletion, reduce Aβ clearance from CNS, which confirms the hypothesis of the astrocytic share of astrocytic water transport in the Aβ level. This condition further leads to the formation of extracellular Aβ aggregates, the accumulation of which contributes to AD progression (Ross et al. 2003). These data were supplemented by Hawkes et al. study that revealed the correlation of APOE genotypes with perivascular drainage disruption of Aβ the adult mouse brain and with changes in cerebrovascular basement membrane protein levels (Hawkes et al. 2012). Aβ distribution in murine arteries, expressing the human *APOE4* gene, was significantly impaired compared to the absence of interference in mice expressing the human *APOE3* gene and wild-type control mice, at both 3- and 16-months of age (Hawkes et al. 2012). Accordingly, APOE4 expression may promote Aβ accumulation in the brain by modifying expression of the cerebral vascular basal membrane proteins in mice.

The long-term high-fat diet (HFD) also causes disturbances in the other components of NVU. Mice on a diet with high fat content have decreased number of pericytes in the brain blood vessels. Both the pericytes and the smooth muscle cells regulate the clearance of Aβ, so any structural and functional changes reduce the efficiency of Aβ removal from the brain (Zlokovic 2008). Patients with the hypercholesterolemia are at increased risk of developing AD and CAA, due to increased vascular Aβ load. The latest study shows that coexisting down-regulation of collagen IV and fibronectin, which impairs the cooperation of the endothelial cells, astrocytes and extracellular matrix, further increase the risk of both these pathological processes (Hawkes et al. 2015). The microvascular changes observed in the human brain correlate with deterioration of the patient’s status in the course of AD. These may result in reduction in the density of microvessels density and decrease of the CBF predisposing the brain to ischemia (Iadecola and Nedergaard 2007).

### Hypercholesterolemia and insulin-dependent metabolic disorders significantly deteriorate the course of neurodegenerative brain diseases

The high-fat diet-induced hypercholesterolemia and abdominal obesity are well documented risk-factors of the cardiovascular diseases, metabolic syndrome (MetS) and cerebral stroke (Stapleton et al. 2008). Moreover, a correlation between above-mentioned risk factors and neurodegenerative diseases have also been found (Stapleton et al. 2008). They increase the risk of oxidative stress and insulin resistance, leading to the hyperinsulinemia. The results of several studies suggest that hyperinsulinemia may deteriorate brain damage and increase cognitive decline or dementia in the course of AD (Milionis et al. 2008; Stapleton et al. 2008).

The results of in vivo studies showed the influence of HFD on the development of the insulin resistance in the CNS (Arnold et al. 2014). Disturbance of the insulin signalling pathways and an increased expression of the serine-phosphorylated insulin receptor substrate 1 (IRS1-pS616), a marker of the insulin resistance, were detected in the hippocampus of the HFD mice (Arnold et al. 2014). The HFD has also been associated with the impairment of the synaptic integrity as well as memory and cognitive functions (Arnold et al. 2014). The experimental evidence shows that both, short-term diet with very high levels of fat and long-term diet with moderate fat content, cause many changes in the insulin signalling pathways and induce insulin resistance in the CNS. One can hypothesize that this may be associated with the chronic hyperactivation of neurons induced by the HFD. It can also be the result of activation and excessive production of inflammatory factors or the presence of ROS and induction of oxidative stress (Arnold et al. 2014).

Interestingly, consumption of water with high content of sucrose not only leads to insulin resistance, but also to hypercholesterolemia in APP/PS1 mice on a standard chow diet (Cao et al. 2007). This points to the important fact that the insulin resistance along with hypercholesterolemia can be independent of the fat intake. The existence of a common mechanisms present in both these diseases creates new opportunities to control fat levels by modulating the levels of glucose, which can be used to control the risk of AD development.

Recent reports suggest that type 2 diabetes mellitus (T2DM) can be another important factor involved in the development of cognitive decline and AD (Milionis et al. 2008). Interestingly, changes in insulin signalling may not only increase the oxidative stress and promote synaptic loss, but also increase the hyperphosphorylation of Tau protein by regulating Tau protein kinases and phosphatases (Bosco et al. 2011). Furthermore, insulin may enhance inflammatory response in the CNS and it can also modulate the level of Aβ by pathways which use proteins neprilysin (NEP) and IDE to the degradation of Aβ (Farris et al. 2003). Considering that insulin has an effect on a number of metabolites and signalling molecules, it is likely that regulation of its level can influence both the levels of cholesterol and the development of neurodegenerative diseases in the brain. So far, in only few studies the relationship between the hypercholesterolemia and the hyperinsulinemia leading to insulin resistance was investigated, but there is a growing interest in the use of the cholesterol-lowering and anti-diabetic drugs for the treatment of patients with AD (Patrone et al. 2014).

In summary, for a long time cholesterol has been regarded as critically important for several physiological and pathological processes in both developing and adult brain. The high interest in the cholesterol metabolism studies may be at least partially explained by its exceptional biochemical properties, in particular, absolute water insolubility. During the recent years, however, studies of the brain cholesterol homeostasis and its dysregulation have acquired a considerable impact caused mainly by linking the cholesterol metabolic dysfunctions with numerous pathological processes such as memory and cognitive impairment, neurodegenerative diseases, stroke as well as other cerebrovascular diseases, and insulin-dependent metabolic disorders. A growing body of evidence suggests that there are even more connections between the hypercholesterolemia and some pathological processes in the brain. New questions still arise and the cholesterol-related pathological mechanisms in the brain require further investigation. The use of cholesterol as a potential therapeutic target requires a thorough investigation of both the individual cell types (the NVU elements) and their iterations in the CNS, and the numerous modulators that determine precise regulation of cholesterol homeostasis in the brain.

### Abbreviations

24S-OHC, 24(S)-hydroxycholesterol; 27-OHC, 27-hydroxycholesterol; 7D, 7-dehydrocholesterol; Aβ, amyloid β; ABC, ATP-binding cassette; acetyl-CoA, acetyl coenzyme A; AD, Alzheimer’s disease; APP, amyloid precursor protein; APOE, apolipoprotein E; AQP4, aquaporin 4; ATP, adenosine triphosphate; BBB, blood brain barrier; BCSFB, blood-cerebrospinal fluid barrier; CAA, cerebral amyloid angiopathy; CBF, cerebral blood flow; CNS, central nervous system; CSF, cerebrospinal fluid; CYP27A1, sterol 27-hydroxylase; CYP46A1, cholesterol 24-hydroxylase; CYP51, lanosterol 14-alpha demethylase; DE, desmosterol; DHCR24, 24-dehydrocholesterol reductase; ER, the endoplasmic reticulum; FGF-1, a fibroblast growth factor 1; ɣGT, ɣ-glutamyl transpeptidase; Glu, glutamate; GSH, glutathione; GSSG, glutathione disulfide; HD, Huntington’s disease; HDL, high-density lipoprotein; HFD, high-fat diet; HMG-CoA reductase, 3-hydroxy-3-methylglutaryl-CoA reductase; IDE, insulin degrading enzyme; IRS1-pS616, serine-phosphorylated insulin receptor substrate 1; LA, lanosterol; LDL, low-density lipoprotein; LDLR, low-density lipoprotein receptor; LRP1, low-density lipoprotein receptor-related protein 1; LT, lathosterol; LXR, liver X receptor; MetS, metabolic syndrome; MMP-9, matrix metalloproteinase-9; NEP, neprilysin; NVU, neurovascular unit; PS1, presenilin 1; ROS, reactive oxygen species; SR-BI, scavenger receptor class B type I; SREBP2, sterol regulatory element-binding protein 2; T2DM, type 2 diabetes mellitus.

## References

[CR1] Abbott NJ, Rönnbäck L, Hansson E (2006). Astrocyte–endothelial interactions at the blood–brain barrier. Nat Rev Neurosci.

[CR2] Abildayeva K, Jansen PJ, Hirsch-Reinshagen V (2006). 24(S)-hydroxycholesterol participates in a liver X receptor-controlled pathway in astrocytes that regulates apolipoprotein E-mediated cholesterol efflux. J Biol Chem.

[CR3] Anderson R, Barnes JC, Bliss TVP (1998). Behavioural, physiological and morphological analysis of a line of apolipoprotein E knockout mouse. Neuroscience.

[CR4] Apelt J, Schliebs R (2001). β-amyloid-induced glial expression of both pro- and anti-inflammatory cytokines in cerebral cortex of aged transgenic Tg2576 mice with Alzheimer plaque pathology. Brain Res.

[CR5] Arnold SE, Lucki I, Brookshire BR (2014). High fat diet produces brain insulin resistance, synaptodendritic abnormalities and altered behavior in mice. Neurobiol Dis.

[CR6] Asahina M, Yoshiyama Y, Hattori T (2001). Expression of matrix metalloproteinase-9 and urinary-type plasminogen activator in Alzheimer’s disease brain. Clin Neuropathol.

[CR7] Aytan N, Jung T, Tamtürk F (2008). Oxidative stress related changes in the brain of hypercholesterolemic rabbits. Biofactors.

[CR8] Balazs Z, Panzenboeck U, Hammer A (2004). Uptake and transport of high-density lipoprotein (HDL) and HDL-associated alpha-tocopherol by an in vitro blood-brain barrier model. J Neurochem.

[CR9] Björkhem I (2006). Crossing the barrier: oxysterols as cholesterol transporters and metabolic modulators in the brain. J Intern Med.

[CR10] Björkhem I, Leoni V, Meaney S (2010). Genetic connections between neurological disorders and cholesterol metabolism. J Lipid Res.

[CR11] Bogdanovic N, Bretillon L, Lund EG (2001). On the turnover of brain cholesterol in patients with Alzheimer’s disease. Abnormal induction of the cholesterol-catabolic enzyme CYP46 in glial cells. Neurosci Lett.

[CR12] Bosco D, Fava A, Plastino M (2011). Possible implications of insulin resistance and glucose metabolism in Alzheimer’s disease pathogenesis. J Cell Mol Med.

[CR13] Brown MS, Goldstein JL (1999). A proteolytic pathway that controls the cholesterol content of membranes, cells, and blood. Proc Natl Acad Sci U S A.

[CR14] Brown J, Theisler C, Silberman S (2004). Differential expression of cholesterol hydroxylases in Alzheimer’s disease. J Biol Chem.

[CR15] Cao D, Lu H, Lewis TL, Li N (2007). Intake of sucrose-sweetened water induces insulin resistance and exacerbates memory deficits and amyloidosis in a transgenic mouse model of Alzheimer disease. J Biol Chem.

[CR16] Castellano JM, Kim J, Stewart FR (2011). Human apoE isoforms differentially regulate brain amyloid-β peptide clearance. Sci Transl Med.

[CR17] Chen YL, Wang LM, Chen Y (2016). Changes in astrocyte functional markers and β-amyloid metabolism-related proteins in the early stages of hypercholesterolemia. Neuroscience.

[CR18] Chen J, Zhang X, Kusumo H (2013). Cholesterol efflux is differentially regulated in neurons and astrocytes: implications for brain cholesterol homeostasis. Biochim Biophys Acta - Mol Cell Biol Lipids.

[CR19] Chung W-S, Verghese PB, Chakraborty C (2016). Novel allele-dependent role for {APOE} in controlling the rate of synapse pruning by astrocytes. Proc Natl Acad Sci U S A.

[CR20] Corder EH, Saunders AM, Strittmatter WJ (1993). Gene dose of apolipoprotein E type 4 allele and the risk of Alzheimer’s disease in late onset families. Science.

[CR21] Crawford A, Fassett RG, Geraghty DP (2012). Relationships between single nucleotide polymorphisms of antioxidant enzymes and disease. Gene.

[CR22] de Oliveira J, Moreira ELG, dos Santos DB (2014). Increased susceptibility to amyloid-β-induced neurotoxicity in mice lacking the low-density lipoprotein receptor. J Alzheimers Dis.

[CR23] Dietschy JM, Turley SD (2004). Thematic review series: brain lipids. Cholesterol metabolism in the central nervous system during early development and in the mature animal. J Lipid Res.

[CR24] Dringen R (2000). Metabolism and functions of glutathione in brain. Prog Neurobiol.

[CR25] Edwards PA, Kennedy MA, Mak PA (2002). LXRs; oxysterol-activated nuclear receptors that regulate genes controlling lipid homeostasis. Vasc Pharmacol.

[CR26] Ehehalt R, Keller P, Haass C (2003). Amyloidogenic processing of the Alzheimer β-amyloid precursor protein depends on lipid rafts. J Cell Biol.

[CR27] Faraco G, Sugiyama Y, Lane D (2016). Perivascular macrophages mediate the neurovascular and cognitive dysfunction associated with hypertension. J Clin Invest.

[CR28] Farris W, Mansourian S, Chang Y (2003). Insulin-degrading enzyme regulates the levels of insulin, amyloid beta-protein, and the beta-amyloid precursor protein intracellular domain in vivo. Proc Natl Acad Sci U S A.

[CR29] Gaylor J (2002). Membrane-bound enzymes of cholesterol synthesis from lanosterol. Biochem Biophys Res Commun.

[CR30] Ghosh S, Wu MD, Shaftel SS (2013). Sustained interleukin-1β overexpression exacerbates tau pathology despite reduced amyloid burden in an Alzheimer’s mouse model. J Neurosci.

[CR31] Ghribi O, Larsen B, Schrag M, Herman MM (2006). High cholesterol content in neurons increases BACE, β-amyloid, and phosphorylated tau levels in rabbit hippocampus. Exp Neurol.

[CR32] Gosselet F, Saint-Pol J, Fenart L (2014). Effects of oxysterols on the blood-brain barrier: implications for Alzheimer’s disease. Biochem Biophys Res Commun.

[CR33] Han X, Chen M, Wang F (2013). Forebrain engraftment by human glial progenitor cells enhances synaptic plasticity and learning in adult mice. Cell Stem Cell.

[CR34] Hawkes CA, Gentleman SM, Nicoll JA, Carare RO (2015). Prenatal high-fat diet alters the cerebrovasculature and clearance of β-amyloid in adult offspring. J Pathol.

[CR35] Hawkes CA, Sullivan PM, Hands S (2012). Disruption of arterial perivascular drainage of amyloid-β from the brains of mice expressing the human APOE ε4 allele. PLoS One.

[CR36] Hayashi H, Campenot RB, Vance DE, Vance JE (2004). Glial lipoproteins stimulate axon growth of central nervous system neurons in compartmented cultures. J Biol Chem.

[CR37] Herz J (2009). Apolipoprotein E receptors in the nervous system. Curr Opin Lipidol.

[CR38] Herzig MC, Winkler DT, Burgermeister P (2004). Aβ is targeted to the vasculature in a mouse model of hereditary cerebral hemorrhage with amyloidosis. Nat Neurosci.

[CR39] Hirrlinger J, König J, Keppler D (2001). The multidrug resistance protein MRP1 mediates the release of glutathione disulfide from rat astrocytes during oxidative stress. J Neurochem.

[CR40] Hirsch-Reinshagen V, Zhou S, Burgess BL (2004). Deficiency of ABCA1 impairs apolipoprotein E metabolism in brain. J Biol Chem.

[CR41] Hohnholt MC, Dringen R (2014). Short time exposure to hydrogen peroxide induces sustained glutathione export from cultured neurons. Free Radic Biol Med.

[CR42] Hughes TM, Kuller LH, Lopez OL (2012). Markers of cholesterol metabolism in the brain show stronger associations with cerebrovascular disease than Alzheimer’s disease. J Alzheimers Dis.

[CR43] Iadecola C, Nedergaard M (2007). Glial regulation of the cerebral microvasculature. Nat Neurosci.

[CR44] Iliff JJ, Wang M, Liao Y, et al (2012) A Paravascular pathway facilitates CSF flow through the brain parenchyma and the clearance of interstitial solutes, Including Amyloid β. Sci Transl Med 4:147ra111. doi:10.1126/scitranslmed.3003748 10.1126/scitranslmed.3003748 PMC3551275 22896675

[CR45] Jansen PJ, Lütjohann D, Thelen KM (2009). Absence of ApoE upregulates murine brain ApoD and ABCA1 levels, but does not affect brain sterol levels, while human ApoE3 and human ApoE4 upregulate brain cholesterol precursor levels. J Alzheimers Dis.

[CR46] Kim WS, Guillemin GJ, Glaros EN (2006). Quantitation of ATP-binding cassette subfamily-a transporter gene expression in primary human brain cells. Neuroreport.

[CR47] Kim WS, Rahmanto AS, Kamili A (2007). Role of ABCG1 and ABCA1 in regulation of neuronal cholesterol efflux to apolipoprotein E discs and suppression of amyloid-β peptide generation. J Biol Chem.

[CR48] Kowiański P, Lietzau G, Steliga A (2013). The astrocytic contribution to neurovascular coupling – still more questions than answers?. Neurosci Res.

[CR49] Lahiri DK (2004). Apolipoprotein E as a target for developing new therapeutics for Alzheimer’s disease based on studies from protein, RNA, and regulatory region of the gene. J Mol Neurosci.

[CR50] Lehtinen MK, Bjornsson CS, Dymecki SM (2013). The choroid plexus and cerebrospinal fluid: emerging roles in development, disease, and therapy. J Neurosci.

[CR51] Levi O, Lütjohann D, Devir A (2005). Regulation of hippocampal cholesterol metabolism by apoE and environmental stimulation. J Neurochem.

[CR52] Liao WL, Heo GY, Dodder NG, et al (2011) Quantification of cholesterol-metabolizing p450s CYP27A1 and CYP46A1 in neural tissues reveals a lack of enzyme-product correlations in human retina but not human brain. J Proteome Res 10:241–248. doi:10.1021/pr1008898 10.1021/pr1008898 PMC3064498 21049985

[CR53] Liu J-P, Tang Y, Zhou S (2010). Cholesterol involvement in the pathogenesis of neurodegenerative diseases. Mol Cell Neurosci.

[CR54] Liu Q, Trotter J, Zhang J (2010). Neuronal LRP1 knockout in adult mice leads to impaired brain lipid metabolism and progressive, age-dependent synapse loss and neurodegeneration. J Neurosci.

[CR55] Lütjohann D, Papassotiropoulos A, Björkhem I (2000). Plasma 24S-hydroxycholesterol (cerebrosterol) is increased in Alzheimer and vascular demented patients. J Lipid Res.

[CR56] Mahley RW, Rall SC (2000). APOLIPOPROTEIN E: far more than a lipid transport protein. Annu Rev Genomics Hum Genet.

[CR57] Martiskainen H, Haapasalo A, Kurkinen KM (2013). Targeting ApoE4/ApoE receptor LRP1 in Alzheimer’s disease. Expert Opin Ther Targets.

[CR58] Mashayekhi F, Hadavi M, Vaziri HR, Naji M (2010). Increased acidic fibroblast growth factor concentrations in the serum and cerebrospinal fluid of patients with Alzheimer’s disease. J Clin Neurosci.

[CR59] Matsuda A, Nagao K, Matsuo M (2013). 24(S)-hydroxycholesterol is actively eliminated from neuronal cells by ABCA1. J Neurochem.

[CR60] Milionis HJ, Florentin M, Giannopoulos S (2008). Metabolic syndrome and Alzheimer’s disease: a link to a vascular hypothesis?. CNS Spectr.

[CR61] Morell P, Jurevics H (1996). Origin of cholesterol in myelin. Neurochem Res.

[CR62] Nagayasu Y, Morita SY, Hayashi H (2014). Increasing cellular level of phosphatidic acid enhances FGF-1 production in long term-cultured rat astrocytes. Brain Res.

[CR63] Nieweg K, Schaller H, Pfrieger FW (2009). Marked differences in cholesterol synthesis between neurons and glial cells from postnatal rats. J Neurochem.

[CR64] Oberheim NA, Takano T, Han X (2009). Uniquely hominid features of adult human astrocytes. J Neurosci.

[CR65] Oram JF, Heinecke JW (2005). ATP-binding cassette transporter A1: a cell cholesterol exporter that protects against cardiovascular disease. Physiol Rev.

[CR66] Orth M, Bellosta S (2012). Cholesterol: its regulation and role in central nervous system disorders. Cholesterol.

[CR67] Papassotiropoulos A, Lutjohann D, Bagli M (2000). Plasma 24S-hydroxycholesterol: a peripheral indicator of neuronal degeneration and potential state marker for Alzheimer’s disease. Neuroreport.

[CR68] Patrone C, Eriksson O, Lindholm D (2014). Diabetes drugs and neurological disorders: new views and therapeutic possibilities. Lancet Diabetes Endocrinol.

[CR69] Pfrieger FW, Ungerer N (2011). Cholesterol metabolism in neurons and astrocytes. Prog Lipid Res.

[CR70] Riddell DR, Zhou H, Comery TA (2007). The LXR agonist TO901317 selectively lowers hippocampal Aβ42 and improves memory in the Tg2576 mouse model of Alzheimer’s disease. Mol Cell Neurosci.

[CR71] Róg T, Stimson LM, Pasenkiewicz-Gierula M (2008). Replacing the cholesterol hydroxyl group with the ketone group facilitates sterol flip-flop and promotes membrane fluidity. J Phys Chem B.

[CR72] Ross GW, O’Callaghan JP, Sharp DS (2003). Quantification of regional glial fibrillary acidic protein levels in Alzheimer’s disease. Acta Neurol Scand.

[CR73] Russell DW, Halford RW, Ramirez DMO (2009). Cholesterol 24-hydroxylase: an enzyme of cholesterol turnover in the brain. Annu Rev Biochem.

[CR74] Saeed AA, Genové G, Li T (2014). Effects of a disrupted blood-brain barrier on cholesterol homeostasis in the brain. J Biol Chem.

[CR75] Sagara J, Makino N, Bannai S (1996). Glutathione efflux from cultured astrocytes. J Neurochem.

[CR76] Saher G, Brügger B, Lappe-Siefke C (2005). High cholesterol level is essential for myelin membrane growth. Nat Neurosci.

[CR77] Shin HK, Jones PB, Garcia-Alloza M (2007). Age-dependent cerebrovascular dysfunction in a transgenic mouse model of cerebral amyloid angiopathy. Brain.

[CR78] Stapleton PA, James ME, Goodwill AG, Frisbee JC (2008). Obesity and vascular dysfunction. Pathophysiology.

[CR79] Strickland DK, Au DT, Cunfer P, Muratoglu SC (2014). Low-density lipoprotein receptor-related protein-1: role in the regulation of vascular integrity. Arterioscler Thromb Vasc Biol.

[CR80] Sucher NJ, Lipton SA (1991). Redox modulatory site of the NMDA receptor-channel complex: regulation by oxidized glutathione. J Neurosci Res.

[CR81] Sun Y, Wu S, Bu G (1998). Glial fibrillary acidic protein-apolipoprotein E (apoE) transgenic mice: astrocyte-specific expression and differing biological effects of astrocyte-secreted apoE3 and apoE4 lipoproteins. J Neurosci.

[CR82] Thirumangalakudi L, Samany PG, Owoso A (2006). Angiogenic proteins are expressed by brain blood vessels in Alzheimer’s disease. J Alzheimers Dis.

[CR83] Torp R, Danbolt NC, Babaie E (1994). Differential expression of two glial glutamate transporters in the rat brain: in situ hybridization study. Eur J Neurosci.

[CR84] Vance JE (2006). Lipid imbalance in the neurological disorder, Niemann-pick C disease. FEBS Lett.

[CR85] Vance JE, Hayashi H (2010). Formation and function of apolipoprotein E-containing lipoproteins in the nervous system. Biochim Biophys Acta - Mol Cell Biol Lipids.

[CR86] Vance JE, Pan D, Campenot RB (1994). Evidence that the major membrane lipids, except cholesterol, are made in axons of cultured rat sympathetic neurons. J Neurochem.

[CR87] Wahrle SE, Jiang H, Parsadanian M (2005). Deletion of Abca1 increases Abeta deposition in the PDAPP transgenic mouse model of Alzheimer disease. J Biol Chem.

[CR88] Wahrle SE, Jiang H, Parsadanian M (2008). Overexpression of ABCA1 reduces amyloid deposition in the PDAPP mouse model of Alzheimer disease. J Clin Invest.

[CR89] Weller RO, Subash M, Preston SD (2008). Clearance of Aβ from the brain in Alzheimer’s disease: perivascular drainage of amyloid-β peptides from the brain and its failure in cerebral amyloid Angiopathy and Alzheimer’s disease: perivascular drainage of Aβ peptides and cerebral amyloid Angiopathy. Brain Pathol.

[CR90] William Rebeck G, Reiter JS, Strickland DK, Hyman BT (1993). Apolipoprotein E in sporadic Alzheimer’s disease: allelic variation and receptor interactions. Neuron.

[CR91] Xiao M, Hu G (2014). Involvement of aquaporin 4 in astrocyte function and neuropsychiatric disorders. CNS Neurosci Ther.

[CR92] Xu PT, Gilbert JR, Qiu HL (1999). Specific regional transcription of apolipoprotein E in human brain neurons. Am J Pathol.

[CR93] Xu Z, Xiao N, Chen Y (2015). Deletion of aquaporin-4 in APP/PS1 mice exacerbates brain Aβ accumulation and memory deficits. Mol Neurodegener.

[CR94] Yan P, Hu X, Song H (2006). Matrix metalloproteinase-9 degrades amyloid-β fibrils in vitro and compact plaques in situ. J Biol Chem.

[CR95] Yao X, Hrabetova S, Nicholson C, Manley GT (2008). Aquaporin-4-deficient mice have increased extracellular space without tortuosity change. J Neurosci.

[CR96] Ye B, Shen H, Zhang J (2015). Dual pathways mediate β-amyloid stimulated glutathione release from astrocytes. Glia.

[CR97] Zambón D, Quintana M, Mata P (2010). Higher incidence of mild cognitive impairment in familial hypercholesterolemia. Am J Med.

[CR98] Zelcer N, Khanlou N, Clare R (2007). Attenuation of neuroinflammation and Alzheimer’s disease pathology by liver x receptors. Proc Natl Acad Sci U S A.

[CR99] Zhang C, Rodriguez C, Spaulding J (2012). Age-dependent and tissue-related glutathione redox status in a mouse model of Alzheimer’s disease. J Alzheimers Dis.

[CR100] Zhang Y, Sloan SA, Clarke LE (2016). Purification and characterization of progenitor and mature human astrocytes reveals transcriptional and functional differences with mouse. Neuron.

[CR101] Zlokovic BV (2008). The blood-brain barrier in health and chronic neurodegenerative disorders. Neuron.

[CR102] Zlokovic BV (2011). Neurovascular pathways to neurodegeneration in Alzheimer’s disease and other disorders. Nat Rev Neurosci.

